# An Ex Vivo Model of Post Infectious Bronchiolitis Obliterans in Children Using Reconstituted Human Bronchial Epithelium

**DOI:** 10.3390/biom16050736

**Published:** 2026-05-18

**Authors:** Julie Mazenq, Léa Moreno, Jean-Christophe Dubus, Fabien Chuette, Louisa Goumidi, Nicoleta Panait, Pascal Chanez, Delphine Gras

**Affiliations:** 1 Pediatric Pulmonology Department and Reference Center for Rare Lung Disease RespiRare, Assistance Publique des Hôpitaux de Marseille (AP-HM), Hôpital Universitaire Timone-Enfants, 13385 Marseille, France; jean-christophe.dubus@ap-hm.fr; 2 Pediatric Pulmonology Department, Aix-Marseille University, INSERM, INRAE, C2VN, 13005 Marseille, France; lea.moreno98@laposte.net (L.M.); fabien.chuette@etu.univ-amu.fr (F.C.); louisa.goumidi@univ-amu.fr (L.G.); pascal.chanez@univ-amu.fr (P.C.); delphine.gras@univ-amu.fr (D.G.); 3 Pediatric Surgery Department, Marseille Public University Hospital System, 13354 Marseille, France; nicoleta.panait@ap-hm.fr; 4 Department of Respiratory Diseases, INSERM C2VN, Aix-Marseille University, 13005 Marseille, France

**Keywords:** post-infectious bronchiolitis obliterans, children, bronchial epithelium, alarmins, interferon

## Abstract

Introduction: Post-infectious bronchiolitis obliterans (PIBO) is a rare and severe chronic lung disease. Our goal was to characterize respiratory epithelium in children with PIBO, which remains unexplored, using an ex vivo model culture. Methods: Proximal bronchial biopsies from children with PIBO and reconstituted bronchial epithelium from PIBO patients (*n* = 3) and controls (n = 17) were analyzed using an air–liquid interface culture model. Epithelial cell composition, barrier integrity, and mediator production, including mucins, inflammatory and antiviral responses, were assessed in this pathological and functional approach. Results: Epithelial thickness was assessed in PIBO biopsies. Ex vivo reconstituted PIBO epithelia appeared to exhibit comparable cohesion and cell composition to controls. Mucin expression and secretion were likewise similar between groups. PIBO epithelial might have displayed reduced IL-33 transcript levels and decreased TSLP secretion, whereas IFN-λ1, IFN-λ2-3 and IFN-β secretion could have been elevated. No differences were detected in remodeling markers (MMP-9 and YKL-40). Conclusions: In summary, ex vivo model of PIBO epithelia suggested that the epithelium may preserve structural characteristics and mucin production, without evidence of remodeling. However, PIBO epithelial cells may have a distinct immune profile, with lower alarmin expression and higher interferon secretion. This could indicate a tendency toward enhanced antiviral response rather than structural changes. These preliminary results need to be confirmed in larger cohorts.

## 1. Introduction

Post-infectious bronchiolitis obliterans (PIBO) is a chronic obstructive pulmonary disease involving inflammation and fibrosis of the distal airways [1,2,3] that leads to the narrowing and/or complete obstruction of the bronchiolar lumen. PIBO is the most common form of bronchiolitis obliterans in children and typically occurs after a severe lower respiratory tract infection in previously healthy individuals [1]. Several infectious agents have been implicated, including adenovirus, *Mycoplasma pneumoniae*, influenza virus, parainfluenza virus, measles virus and varicella virus [4,5,6,7].

PIBO is a rare but severe condition that can lead to persistent respiratory symptoms and progressive respiratory insufficiency over time. Most epidemiological data have been derived from cohorts in South America and Korea [8]. However, recent European data, including a French cohort study [7], have highlighted the heterogeneity in disease trajectories according to age. Notably, younger children tend to experience more severe initial infections and exhibit distinct viral profiles. Diagnostic criteria are based on international consensus and include a history of preceding respiratory infection, persistent and irreversible airway obstruction and characteristic small airway abnormalities on thoracic computed tomography (CT), such as mosaic attenuation and bronchial wall thickening. Bronchiectasis may also be observed in some cases. Other chronic pulmonary diseases must be excluded [1,2,8,9].

Despite advances in diagnostic approaches, there are currently no standardized long-term management or treatment guidelines for PIBO. Therapeutic strategies remain heterogeneous and largely empirical, reflecting the limited understanding of disease mechanisms. Treatments have included inhaled and systemic corticosteroids, high-dose intravenous steroids, azithromycin, montelukast, acetylcysteine and supplemental oxygen for hypoxemia [6,7,10,11]. Although early diagnosis and anti-inflammatory treatment can reduce mortality and airway fibrosis, clinical outcomes are still highly variable.

The respiratory epithelium is the primary interface between the host and its environment. It plays a central role in regulating airway inflammation, immune responses and tissue repair [12]. Growing evidence suggests that epithelial injury caused by viral infection, together with the epithelial immune response, critically influences the balance between effective repair and abnormal healing, leading to permanent structural changes. Dysregulation of epithelial processes, such as apoptosis and epithelial–mesenchymal transition, may lead to persistent inflammation, fibrosis and irreversible airway obstruction. Airway remodeling is a hallmark of PIBO [13,14]. However, the specific contribution of the bronchial epithelium at the onset of the disease, particularly in children, remains poorly characterized. We hypothesized that bronchial epithelial injury and altered repair mechanisms are present at the time of PIBO diagnosis and contribute to early airway remodeling [15].

In this study, we aimed to better describe the bronchial epithelium in children at the time of PIBO diagnosis. For this purpose, we investigated morphologic and functional properties of the epithelial tissue to assess differences associated with PIBO using both ex vivo and in vitro analysis. Our findings reveal early functional, but not morphological, bronchial epithelial alterations at diagnosis, which supports the potential role of epithelial dysfunction in the pathogenesis of PIBO.

## 2. Materials and Methods

### 2.1. Study Population and Sample Collection

Children diagnosed with PIBO were enrolled prospectively at the time of diagnosis. Bronchial biopsy samples were obtained during flexible bronchoscopy as clinically indicated. The Institutional Ethics Committee approved this study (approval number: 023-A01544-41), and it was registered with ClinicalTrials.gov (NCT06140901). Written informed consent was obtained from all parents or legal guardians.

Lung tissue samples were collected from children undergoing lung lobectomy for congenital pulmonary airway malformation (CPAM), in collaboration with the Department of Pediatric Surgery, at La Timone-Enfants Hospital in Marseille, France. The samples used in the study were collected from healthy tissue located away from the malformation and served as the control group. The control sample collection protocol was approved by the Ethics Committee for Clinical Research in Thoracic and Cardiovascular Surgery (CERC-SFCTCV-2023-11-21_31945).

Demographic characteristics, including age, sex and diagnosis delay (defined as the time interval between the suspected infectious event and the diagnosis established at the time of chest CT), are reported (Table 1).

### 2.2. Immunohistochemistry

A single biopsy was obtained from each patient at the time of PIBO diagnosis. The biopsy was fixed in 4% neutral-buffered formaldehyde and paraffin-embedded. Five to ten sections per specimen were stained with hematoxylin and eosin.

Digital slides were analyzed using NZViewMD software (Ver. 1.0, Rev. 2) (Hamamatsu Photonics K.K., Hamamatsu, Japan). All analyzable epithelial areas were included in the analysis. Regions with severe artifacts, such as folding, tearing or complete epithelial loss, were excluded if they prevented a reliable morphometric assessment. Otherwise, all remaining evaluable epithelial regions were analyzed.

For each section, the epithelial regions of interest were manually delineated. The epithelial surface area and the length of the basement membrane were measured using a manual freehand line tool that followed the contour of the basement membrane. Epithelial thickness was calculated as the ratio of epithelial area to basement membrane length.

Multiple measurements were performed across all sections for each biopsy (five to ten sections per patient), and all individual measurements were pooled. The final value reported per patient corresponds to the median of all measurements obtained across sections.

### 2.3. Primary Human Bronchial Epithelial Cell Culture

Primary human bronchial epithelial cells (HBECs) were isolated from bronchial biopsies (PIBO patients) or lung tissue (controls). The cells were suspended in Pneumacult™-Ex+ (StemCell Technologies, Inc., Vancouver, BC, Canada) and expanded as monolayers. After expansion, the cells were seeded onto uncoated nucleopore membranes (12 mm diameter, 0.4 μm pore size; Transwell^®^ Clear, Corning Incorporated, Corning, NY, USA). Air–liquid interface (ALI) conditions were established by removing the apical medium and maintaining Pneumacult™-ALI medium (StemCell Technologies, Inc.) in the basolateral compartment. The cells were cultured at 37 °C in a humidified atmosphere with 5% CO_2_ for 28 days to allow differentiation into a polarized mucociliary epithelium.

### 2.4. Epithelial Barrier Integrity Measurement

Transepithelial electrical resistance (TEER) was measured using a dedicated volt-ohm meter (EVOM2, World Precision Instruments, Aston, UK) and expressed in ohms per centimeter (Ω·cm^−^^2^).

### 2.5. Real-Time Quantitative PCR (RT-qPCR) Analysis of Gene Expression

HBECs were collected in RLT buffer (Qiagen), and total RNA was extracted using the RNeasy Mini Kit (Qiagen, Hilden, Germany) according to the manufacturer’s instructions. cDNA was synthesized from the extracted RNA. Quantitative real-time PCR (qPCR) was performed using SYBR Green and gene-specific primers targeting mucins (*MUC5AC* and *MUC5B*), inflammatory markers (*IL-8*), alarmins (*IL-33* and *thymic stromal lymphopoietin* (*TSLP*)) and the antiviral mediator (*IP-10*) (Eurofins Genomics, Nantes, France). Relative gene expression was calculated using the 2^−ΔCt^ method with 18S as the reference gene and was expressed in arbitrary units (A.U.).

### 2.6. Measurement of Mucin Secretion and Inflammatory, Antiviral and Remodeling Mediators

MUC5AC and MUC5B mucin secretion was assessed by indirect ELISA using apical washes as previously described (Singanayagam A, 2022, JCI^16^). Briefly, samples were coated onto plates using carbonate–bicarbonate buffer (sigma-aldrich, Saint Louis, MO, USA) and incubated at 37 °C until complete evaporation. After washing, the wells were blocked with PBS containing 2% BSA and 0.05% Tween-20 and then incubated with primary antibodies against MUC5AC or MUC5B (Invitrogen, Thermo Fisher Scientific, Waltham, MA, USA), followed by washing and incubation with a goat anti-mouse horseradish peroxidase (HRP)-conjugated secondary antibody (Santa Cruz Biotechnology, Dallas, TX, USA). Detection was performed using an HRP substrate (TMB; Thermo Fisher Scientific, Waltham, MA, USA). Absorbance was measured at 450 nm, with background correction at 540 nm, using an EnSight multimode plate reader (PerkinElmer, Shelton, CT, USA).

IL-8 and TSLP release was quantified in basolateral culture supernatants using sandwich ELISA MAX™ kits (BioLegend, San Diego, CA, USA) according to the manufacturer’s instructions.

Other secreted proteins (IFNλ1, IFNλ2-3, IFNβ, IFNα2, IL-33, IP-10, YKL-40, matrix metalloproteinase-9 (MMP-9)) in the culture supernatant were quantified by flow cytometry (Cytoflex LX) using LegendPlex^TM^ (BioLegend, San Diego, CA, USA, custom human panels) according to the manufacturer’s instructions.

### 2.7. Immunostaining

Immunofluorescence staining was performed on cytocentrifugated cells obtained from differentiated ALI cultures. The cells were fixed with acetone-methanol, blocked and permeabilized with PBS containing 2% BSA and 0.05% Tween-20. The cells were then incubated overnight at 4 °C with the following primary antibodies: rabbit monoclonal anti-cytokeratin 5 (CK5) (Cell Signaling, Danvers, MA, USA), rabbit monoclonal anti-α-tubulin (Cell Signaling), rabbit monoclonal anti-MUC5AC (Cell Signaling) and mouse anti-monoclonal anti-chromogranin (Abcam, Cambridge, UK). Secondary labeling was performed for one hour at room temperature using either Alexa Fluor^®^ 488 (Cell Signaling) or Alexa Fluor^®^ 555 (Cell Signaling), followed by DAPI nuclear staining (Sigma-Aldrich, St. Louis, MO, USA, D8417-1MG). The cells were visualized with a Nikon Eclipse Ni microscope. A cell count was performed for each patient and for each target by counting 300 cells per slide at 400× magnification using NIS-Elements BR software (version 4.30.1017).

### 2.8. Statistical Analysis

Unpaired groups were compared using the Mann–Whitney test. Statistical analyses were conducted using GraphPad Prism, version 10.5.2. A *p*-value of less than 0.05 was considered statistically significant.

## 3. Results

### 3.1. Demographic Data

A total of three children with PIBO and 17 controls were included in the study. Demographic characteristics are summarized in Table 1. The median age at diagnosis was similar between the two groups (13 months for the PIBO group vs. 11 months for the control group), and there was no significant difference in median birth weight. Both groups showed a female predominance. There was no difference in neonatal respiratory history between the two groups. Importantly, none of the patients in either group had received systemic corticosteroids or ventilatory support prior to endoscopy. The median diagnostic delay for PIBO was 5 months. No viral infection was detected at the time of bronchoscopy. Adenovirus infection was identified as the suspected trigger in two PIBO cases, while the causative virus remained unidentified in one patient. All PIBO patients had received inhaled corticosteroids prior to diagnosis and systemic corticosteroids following diagnostic bronchoscopy.

### 3.2. Ex Vivo Epithelium Characteristics of Biopsies in PIBO

**Figure 1:**
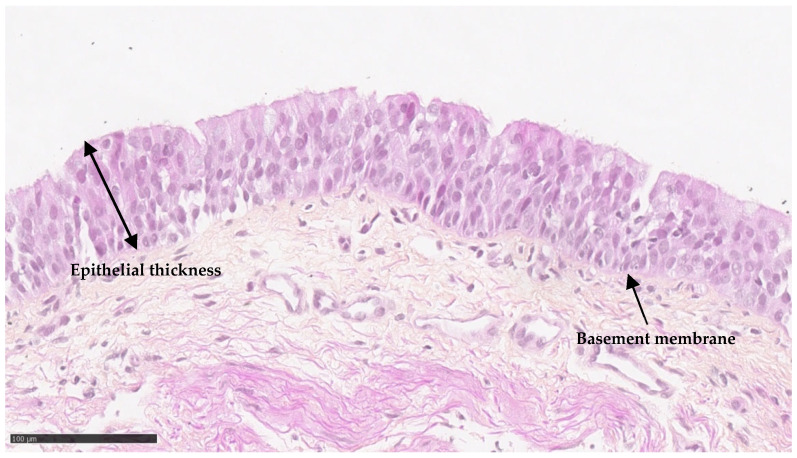
Ex vivo epithelium characteristics of biopsies in PIBO (n = 3). Representative image of bronchial epithelium at the time of PIBO diagnosis (hematoxylin and eosin staining).

Samples from PIBO patients exhibited an epithelial area with a median of 9.6 μm^2^. The median thickness of the epithelium was 23 μm (Figure 1 and Table 2).

### 3.3. Comparable Epithelial Integrity and Cellular Composition in PIBO and CPAM in Ex Vivo Culture Model

**Figure 2:**
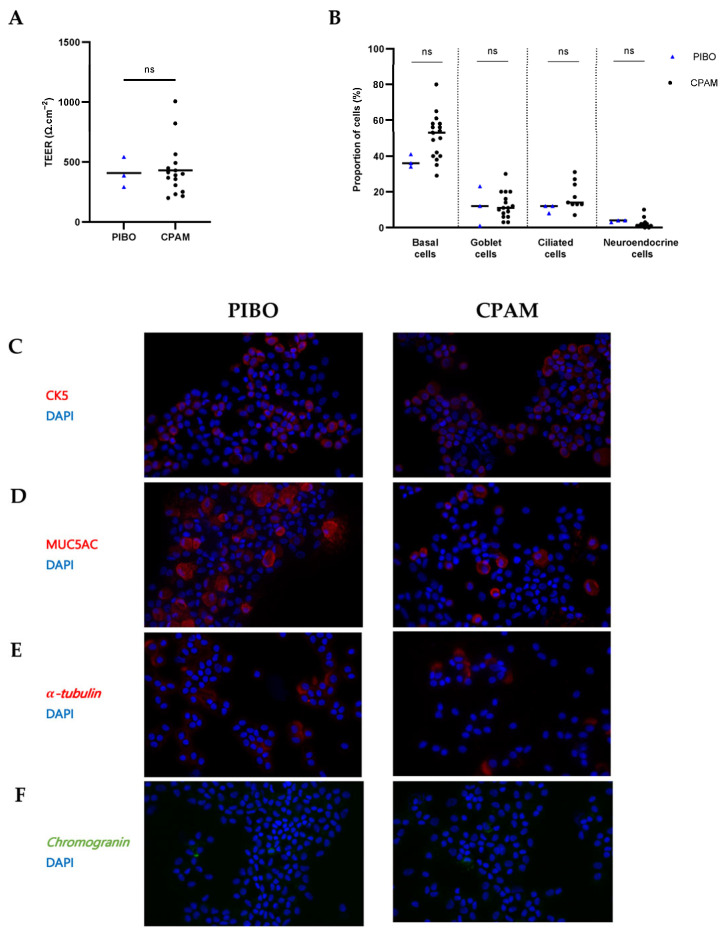
Ex vivo epithelium model: morphological characterization in PIBO (n = 3) compared to controls (n = 17). (**A**) Epithelial integrity was assessed by transepithelial electrical resistance (TEER) measurement. Results are expressed in medians (Ω·cm^−2^). (**B**) Cell proportion of basal cells (CK5-positive), ciliated cells (α-tubulin-positive), goblet cells (MUC5AC-positive) and neuroendocrine cells (chromogranin-positive). Results are expressed as % and represented by the median number of positive cells/total nuclei per field. (**C**–**F**) Representative immunofluorescence of epithelial cell subtype staining on cytocentrifuged cells from PIBO patients and control subjects at 400× magnification. (**C**) Basal cells (CK5). (**D**) Goblet cell (MUC5AC), (**E**) Ciliated cells (α-tubulin) and (**F**) neuroendocrine cells (chromogranin). Nuclei were counterstained with DAPI. ns: not significant (Mann–Whitney U test).

Ex vivo reconstituted PIBO epithelia were successfully obtained. The rate of culture failure was similar for both the PIBO and CPAM samples. There was no increased proportion of non-growing samples in the PIBO group. Epithelial cohesion, as measured indirectly by TEER, was comparable between groups (406.3 ± 127.1 Ω·cm^−2^ in PIBO versus 430.5 ± 216.8 Ω·cm^2^ in CPAM; *p* > 0.99) (Figure 2A).

Analysis of cell composition revealed no significant differences between PIBO and control groups in the proportion of basal cells (CK5-positive; 38.5% vs. 53%, respectively), ciliated cells (α-tubulin-positive; 12% vs. 14%, respectively), mucus-producing cells (MUC5AC-positive; 8.5% vs. 11%, respectively), or neuroendocrine cells (chromogranin-positive; 3.5% vs. 1%, respectively) (Figure 2B–F).

### 3.4. Altered Epithelial Immune and Antiviral Functions in PIBO Ex Vivo Culture Model

**Figure 3:**
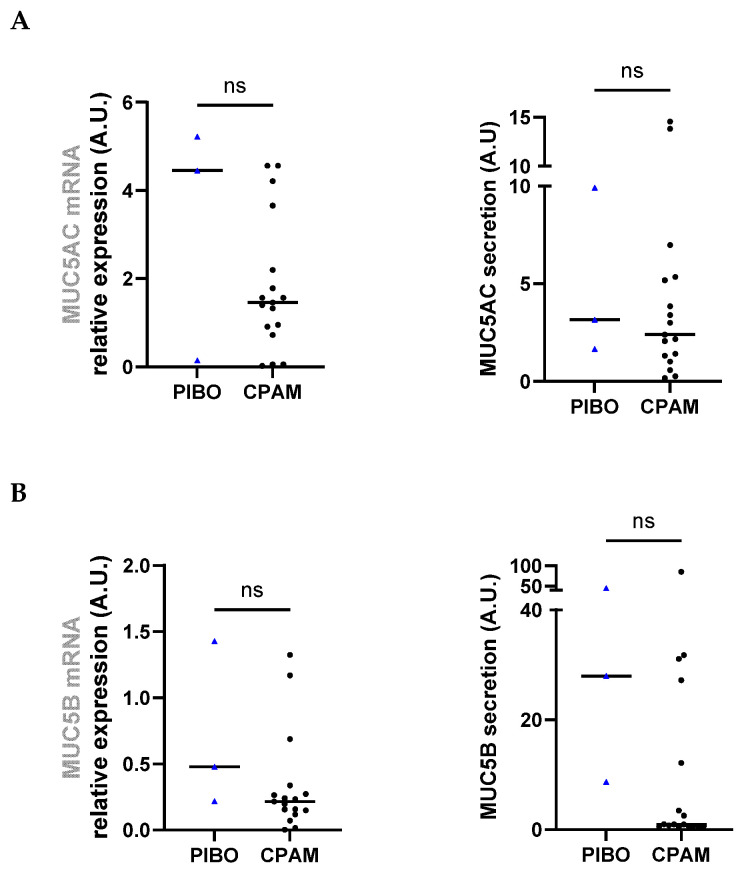
Ex vivo epithelium model functional characterization: mucins analysis in PIBO (blue triangle) (n = 3) compared to controls (black dots) (n = 17). Gene expression and protein release were measured in HBECs and in secretion from apical compartment of the ex vivo culture model, respectively, (**A**) MUC5AC and (**B**) MUC5B. Data are presented as median values in arbitrary units (A.U.). ns: not significant (Mann–Whitney U test).

Analysis of MUC5AC gene expression and secretion levels revealed no significant differences between groups (3.28 ± 2.74 vs. 1.83 ± 1.53 and 4.91 ± 4.40 vs. 3.97 ± 4.29, respectively) (Figure 3A). *MUC5B* gene expression and secretion levels were also comparable between PIBO and the control group (0.71 ± 0.64 vs. 0.33 ± 0.38 and 27.28 ± 18.25 vs. 11.80 ± 22.05, respectively) although a trend toward higher MUC5B secretion was observed in PIBO samples (Figure 3B).

**Figure 4:**
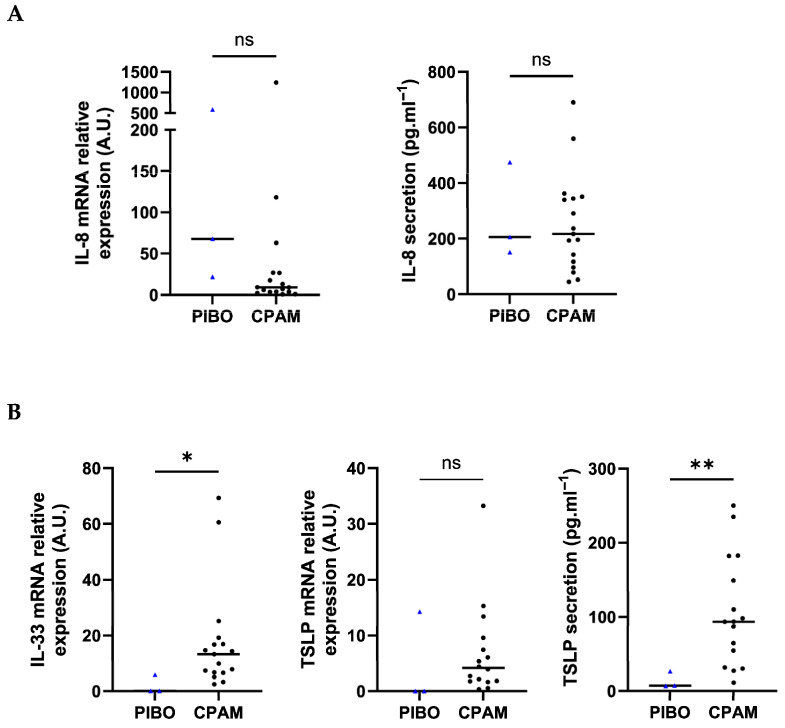
Ex vivo epithelium model functional characterization: inflammatory mediators and alarmins analysis in PIBO (blue triangle) (n = 3) compared to controls (black dots) (n = 17). Gene expression and protein release were measured in HBECs and in secretion from basolateral compartment of the ex vivo culture model, respectively, (**A**) IL-8, (**B**) IL-33 and TSLP. Relative gene expression was expressed in arbitrary units (A.U) and protein secretion in pg·mL^−1^. Data are presented as median values. ns: not significant; * *p* < 0.05; ** *p* < 0.01 (Mann–Whitney U test).

Assessment of inflammatory marker showed that *IL-8* transcript levels tended to be higher in PIBO HBECs than in control HBECs (225.9 ± 314.6 vs. 91.19 ± 297.3, respectively; *p* = 0.07). Protein secretion analysis showed comparable IL-8 levels (277.2 pg·mL^−1^ ± 173.6 vs. 253.6 pg·mL^−1^ ± 177.2, respectively) (Figure 4A).

Beyond classical pro-inflammatory markers, *IL-33* transcript expression was significantly lower in PIBO samples (2.03 ± 3.34 vs. 17.64 ± 18.87, respectively; *p* = 0.02). *TSLP* transcript levels were lower in PIBO cells (4.77 ± 8.23 vs. 6.87 ± 8.3, respectively), though not statistically significantly (Figure 4B). No difference was observed between groups for either *TSLP* isoform, i.e., short-form (sf-TSLP) and long-form (lf-TSLP) (Figure S1). IL-33 secretion was below the detection limit in both groups. Conversely, TSLP secretion was significantly lower in PIBO epithelial cells (13.66 pg·mL^−1^ ± 11.23 vs. 106.3 pg·mL^−1^ ± 74.06, respectively; *p* = 0.04) (Figure 4B).

**Figure 5:**
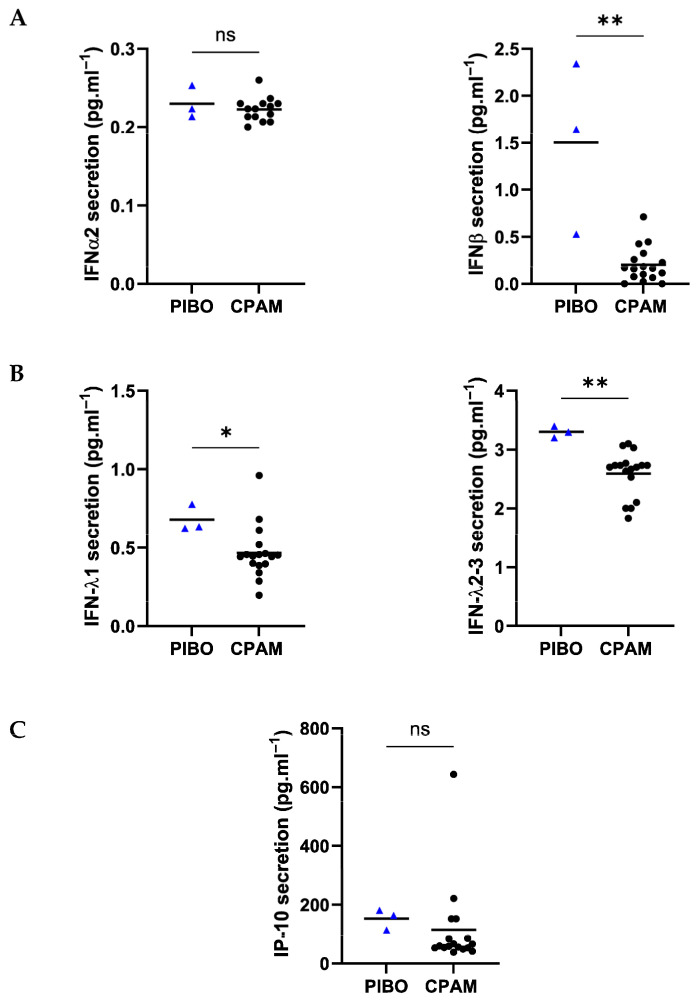
Ex vivo epithelium model functional characterization: antiviral mediators analysis in PIBO (blue triangle) (n = 3) compared to controls (black dots) (n = 17). (**A**) Interferon type I (IFN-α and β), (**B**) type III (IFNλ1 and λ2-3) and (**C**) IP-10 were measured in secretion from basolateral compartment of the ex vivo culture model. Data are presented as median values and are expressed in pg·mL^−1^. ns: not significant; * *p* < 0.05; ** *p* < 0.01 (Mann–Whitney U test).

Antiviral response marker analysis demonstrated significantly higher secretion of IFN-λ1 (0.68 pg·mL^−1^ ± 0.09 vs. 0.47 pg·mL^−1^ ± 0.17, respectively; *p* = 0.03), IFN-λ2-3 (3.3 pg·mL^−1^ ± 0.1 vs. 2.6 pg·mL^−1^ ± 0.38, respectively; *p* = 0.002) and IFN-β (1.5 pg·mL^−1^ ± 0.91 vs. 0.20 pg·mL^−1^ ± 0.18, respectively; *p* = 0.004) in the PIBO samples compared to control samples. However, the secretion levels of IFNα2 (0.23 pg·mL^−1^ ± 0.02 vs. 0.22 pg·mL^−1^ ± 0.02, respectively) and IP-10 (152.7 pg·mL^−1^ ± 34.87 vs. 1114 pg·mL^−1^ ± 145.1, respectively) were not different (Figure 5).

**Figure 6:**
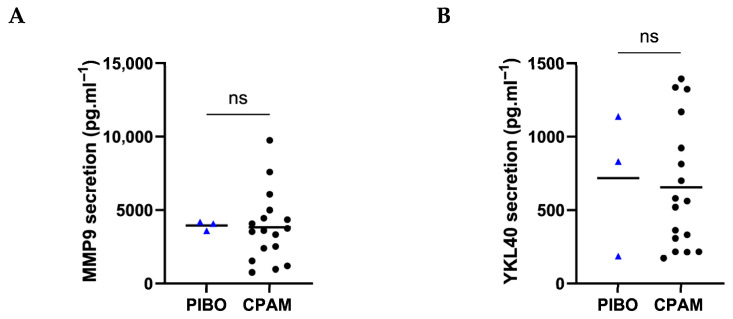
Ex vivo epithelium model functional characterization: remodeling factors analysis in PIBO (blue triangle) (n = 3) compared to controls (black dots) (n = 17). (**A**) MMP9 and (**B**) YKL40 were measured in secretion from basolateral compartment of the ex vivo culture model. Data are presented as median values and are expressed in pg·mL^−1^. ns: not significant; (Mann–Whitney U test).

Remodeling markers, i.e., MMP-9 and YKL40, were comparable between PIBO and the control group (3948 ± 317.9 pg·mL^−1^ vs. 3813 ± 2375 pg·mL^−1^ and 718.3 ± 485.8 pg·mL^−1^ vs. 655.2 ± 431.9 pg·mL^−1^, respectively) (Figure 6).

## 4. Discussion

This study allows for developing ex vivo culture model of bronchial epithelium derived from respiratory epithelial cells obtained from children at the time of PIBO diagnosis and comparing them with control epithelia. Importantly, epithelial samples were obtained several months after the initial infectious insult and therefore likely reflect a chronic or established disease state rather than acute injury or early repair processes. Morphological analyses did not suggest significant differences compared to those of age-matched control children. However, the functional characterization of these cultures may reveal distinct epithelial alterations, including reduced alarmin expression at the time of PIBO diagnosis. Furthermore, patient-derived samples exhibited a potential specific IFN-mediated inflammatory signature in the PIBO epithelium.

To our knowledge, this is the first report describing an ex vivo model of PIBO epithelium. As the interface between the host and the environment, accumulating evidence supports the pivotal role of respiratory epithelium in orchestrating the balance between tissue repair and inflammation. To date, studies in PIBO have primarily focused on adenoviral receptors expressed by epithelial cells [17,18,19]. However, the bronchial epithelium itself has not yet been investigated in the context of this disease. However, insights can be drawn from other chronic inflammatory airway diseases, particularly severe asthma. In this context, the ALI model has been validated; previous studies have demonstrated that the epithelial abnormalities associated with severe asthma persist when bronchial epithelial cells are cultured in vitro and differentiated into a fully mature airway epithelium. These findings suggest that the severe asthma phenotype is an intrinsic property of the bronchial epithelium [12,20].

To characterize the bronchial epithelium in PIBO, we first assessed ex vivo epithelium thickness from PIBO patient biopsies. To date, no data are available in this condition, while evidence from pediatric samples remains scarce and largely limited to the evaluation of epithelial integrity and basement membrane thickness, mostly in asthma [21,22]. Epithelial thickness measurements were performed using a standardized protocol, although variability was observed within individual biopsies, and no equivalent data were available for the control group due to the lack of ethically accessible healthy pediatric bronchial tissue, which limits comparative interpretation.

Development of an ex vivo model of PIBO epithelium enabled a morphological and functional study of this tissue. First, epithelial integrity and cellular composition analysis showed no difference between PIBO and the control group, implying that the structural organization of the bronchial epithelium in PIBO may be similar to that of the pediatric control epithelium, despite the underlying disease. Interestingly, these findings may also suggest that PIBO-derived epithelial cells retain a preserved capacity to regenerate and reconstitute a differentiated airway epithelium under ex vivo conditions. The comparable success rate of ALI cultures and similar TEER values argues against a major intrinsic defect in epithelial proliferation or differentiation in PIBO samples. These results support the hypothesis that epithelial alterations in PIBO may be driven more by the in vivo microenvironment (e.g., inflammation, fibrosis or altered epithelial–mesenchymal interactions) than by a primary defect in epithelial regenerative potential. Because TEER reflects overall tissue cohesion, a more in-depth evaluation of intercellular junctions, using ZO-1 or E-cadherin staining, for example, would help identify subtle alterations in barrier integrity [23,24]. The absence of differences in bronchial epithelial cellular composition has also been reported in other forms of bronchiolitis obliterans, including those occurring after bone marrow transplantation or lung transplantation [25,26]. Then, mucin production did not differ in the PIBO group, either at the transcriptional level or in terms of protein secretion. This disease sequelae clinically leads to bronchial obstruction without mucus hypersecretion. This contrasts with pediatric asthma, for which increased MUC5AC concentrations and an altered MUC5B:MUC5AC ratio have been reported, particularly during acute exacerbations in ALI cultures [22] or sputum [27].

Having established that the structural epithelial architecture is preserved in PIBO, we examined its functional profile, focusing specifically on alarmins, to assess whether a persistent epithelial imprint could be detected at baseline despite the absence of ongoing stimulation. Alarmins are the earliest epithelial danger signals and have not been studied in post-infectious sequelae. They have been well characterized in other chronic respiratory diseases, such as asthma [28]. As key mediators of innate and adaptive immune responses in the airway epithelium, alarmins, such as TSLP and IL-33, are essential for initiating danger signals and orchestrating the immune response to epithelial injury [29,30]. We found reduced expression of TSLP and IL-33 in our study in PIBO-derived epithelial cultures. This may reflect an altered epithelial alarmin response in children with PIBO in an established disease state. However, given the cross-sectional design and the timing of sampling several months after the initial infectious insult, it is not possible to determine whether this decrease reflects altered acute-phase alarmin release, a downstream regulatory mechanism, or long-term epithelial reprogramming. We also explored the two TSLP isoforms, which arise from distinct promoter regions and have different biological roles: the short form (sf-TSLP), mainly associated with epithelial homeostasis, and the long form (lf-TSLP), which is linked to inflammatory response [31,32]. No significant difference in gene expression of either isoform was observed between groups. However, TSLP isoform secretion could not be assessed, as no specific assays are currently available to distinguish between the two isoforms; therefore, our analysis was limited to gene expression.

Beyond these upstream danger signals, we investigated the epithelial inflammatory profile and potential downstream immune pathways of the PIBO epithelium. We focused on IFN types I and III, IL-8, and IP-10 as key mediators of epithelial defense against viral injury. In our model, we observed a potential increase in IFN-related pathway activity markers in PIBO. IFNs are key antiviral mediators and can regulate epithelial immune response [33]. However, in the context of this ex vivo model, their functional impact on epithelial repair or inflammation cannot be directly inferred and would require further investigation, particularly under stimulated conditions. No significant differences were detected in IL-8 and IP-10 protein secretion. These findings suggest that, under basal ex vivo conditions, epithelial inflammatory signaling is only partially altered in PIBO, with no evidence of enhanced neutrophil-associated signaling based on IL-8 levels. While neutrophilic inflammation has been described in PIBO patients in vivo [7,34,35], our data do not support a direct epithelial contribution to this pathway in the absence of stimulation. Notably, increased IL-8 levels have previously been detected in blood and sputum samples from children with PIBO [36,37]. However, this systemic and luminal signal likely reflects inflammatory sources beyond the epithelium, consistent with the absence of IL-8 difference observed in our epithelial model. Beyond inflammation, excessive or prolonged IFN signaling has been shown to impair epithelial proliferation and differentiation, thereby disrupting epithelial repair after viral injury [38]. However, in the context of this ex vivo study, such mechanisms cannot be directly inferred and require validation in longitudinal models.

If sustained IFN signaling impairs epithelial repair, downstream effects on airway remodeling would be anticipated. We therefore examined two remodeling-related proteins, YKL-40 and MMP-9, as potential markers of structural airway changes. In our model, no significant differences were observed between PIBO and controls for either marker. Accordingly, our data do not support the presence of epithelial remodeling alterations involving YKL-40 or MMP-9 under basal conditions. A recent study investigated whether serum YKL-40 levels could serve as a biomarker to distinguish acute exacerbations of PIBO from acute bronchiolitis in young children. Serum concentrations were significantly higher in children admitted with PIBO exacerbations and were positively correlated with disease severity [39]. MMP-9 is a recognized mediator of airway remodeling in chronic airway diseases [40], but its specific role in PIBO has not yet been investigated. Our data do not provide evidence of altered epithelial MMP-9 secretion in this context. This may reflect the timing of sampling, as bronchial epithelial measurements were obtained at a single time point in children with established disease. Therefore, remodeling-related epithelial alterations were not detected in this ex vivo epithelial model under the conditions assessed.

This study has several limitations. First, the small number of PIBO cases (n = 3) limits statistical power and the generalizability of the findings. However, only children who were evaluated at the time of PIBO diagnosis and who showed no evidence of viral infection during bronchoscopy were included. This ensures that the epithelial findings are attributable to PIBO rather than an ongoing infection. Second, substantial interindividual variability was observed, and the small sample size precludes definitive conclusions. Third, while the ex vivo epithelial culture model reconstructs the bronchial epithelium, it does not incorporate immune components that may play an important role in PIBO pathophysiology. Therefore, analyses integrating innate and adaptive immune pathways across different levels of disease severity may provide further insight into epithelial-driven mechanisms of chronic airway inflammation. Another limitation is the sampling site. PIBO primarily affects the distal airway. However, bronchial biopsies were obtained from the proximal airways due to constraints related to endoscopic accessibility. Consequently, the epithelial features described here may not accurately reflect bronchiolar pathology. It is possible that the preserved epithelial structure and function observed in our model reflect the relative preservation of the proximal airways rather than an absence of alterations in the distal compartments. Nevertheless, the proximal airway epithelium remains relevant because it participates in host defense and epithelial–immune interactions. It may also exhibit disease-related alterations or persistent epithelial imprinting following a previous viral infection. Finally, the control group consisted of children with CPAM. Although the bronchial tissue was collected away from the malformed parenchyma and the control group was relatively young (median age of 11 months), these samples do not strictly represent healthy pediatric epithelium. Nevertheless, CPAM-derived epithelial explants have been used in previous pediatric airway epithelial studies because they provide access to non-diseased bronchial epithelium without the need for additional invasive procedures. Within the parameters assessed in this study, these samples did not display features consistent with an inflammatory epithelial phenotype. A recent translational study by Azar et al. showed that molecular alterations in CPAM are largely confined to the malformed tissue and are not detected in adjacent non-cystic bronchial tissue used as controls [41]. Long-term follow-up further supported preserved lung function in these patients. Additionally, the reported molecular abnormalities primarily involve mesenchymal compartments, whereas our study focuses on bronchial epithelial cells. Overall, these data support the use of CPAM-adjacent bronchial tissue as a relevant control in pediatric airway epithelial studies, although a residual microenvironment influence cannot be fully excluded.

## 5. Conclusions

In summary, this translational study focuses on children with a rare chronic lung disease for which pathophysiological studies are scarce and uses an ex vivo model to recreate pathological epithelial tissue. Our results suggest that children with PIBO have low levels of alarmins at the time of diagnosis, which are associated with an IFN-mediated signature. This may reflect an impaired response to the initial viral infection. These findings could serve as an important reference point for further investigations into the airway epithelium of patients with PIBO.

## Figures and Tables

**Table 1:** Clinical characteristics of children at the time of post-infectious bronchiolitis obliterans (PIBO) diagnosis and of children with congenital pulmonary airway malformation (CPAM).

	PIBO Patient 1	PIBO Patient 2	PIBO Patient 3	CPAM (n = 17)	*p*-Value
Sex, (% males)	M	F	F	5 (29% males)	ns
Age at diagnostic (months)	13	6	17	11 (1–192)	ns
Birth weight (g)	3150	3430	3120	3340 (2090–3930)	ns
Surgery					
Lobectomy	NA	NA	NA	13 (76%)	
Segmentectomy	NA	NA	NA	4 (24%)	
Suspected trigger					
Adenovirus	1	0	1	NA	
Unidentified	0	1	0	NA	
Bronchoalveolar lavage (BAL)					
Total BAL cellularity (cells/mm^3^)	760	1010	340	NA	
BAL Neutrophils, (cells/mm^3^)	182	646	17	NA	
Treatment before bronchoscopy					
Inhaled corticosteroid and long-acting β2-agonist	1	1	1	0	
Azithromycin	0	0	0	1 (6%)	

Continuous variables are presented as individual values for PIBO patients and as median (min–max) for the CPAM group. Categorical variables are presented as counts and percentages for the CPAM group and as individual values for PIBO patients. Comparisons were made by using the Mann–Whitney test. NA, not applicable; ns, not significant.

**Table 2:** Quantitative analysis of epithelial thickness in bronchial biopsies from patients with PIBO (n = 3).

Patient	Epithelium Thickness (μm)	Min–Max (μm)
1	51	21-114
2	23	19-71
3	15	8-48

Epithelium thickness was calculated as epithelial area divided by basement membrane length. Values are expressed as the median (min-max) of multiple measurements performed on each biopsy. Measurements were performed on digitized slides using NZViewMD software (Hamamatsu Photonics K.K., Hamamatsu, Japan).

## Data Availability

All data generated or analyzed during this study are included in this article and its Supplementary Materials Files. Further enquiries can be directed to the corresponding author.

## Supplementary Materials

The following supporting information can be downloaded at: https://www.mdpi.com/article/10.3390/biom16050736/s1, Figure S1: Ex vivo epithelium model functional characterization: analysis of alarmin isoforms in PIBO (blue dots) (n = 3) compared to controls (black dots) (n = 17).

## Abbreviations

PIBO	post-infectious bronchiolitis obliterans
CT	computed tomography
CPAM	congenital pulmonary airway malformation
HBECs	primary human bronchial epithelial cells
ALI	air-liquid interface
TEER	transepithelial electrical resistance
RT-qPCR	real-time quantitative PCR
TSLP	thymic stromal lymphopoietin
HRP	horseradish peroxidase
MMP-9	matrix metalloproteinase-9
CK5	cytokeratin 5
