# Supplementary material for: An Ex Vivo Model of Post Infectious Bronchiolitis Obliterans in Children Using Reconstituted Human Bronchial Epithelium
Source: Biomolecules. 2026 May 18;16(5):736. doi: 10.3390/biom16050736 (PMC13205076; doi:10.3390/biom16050736)
Supplement: Supplementary file 1 [file biomolecules-4263427-supplementary.pdf › biomolecules-4263427-supplementary.pdf]

A

B

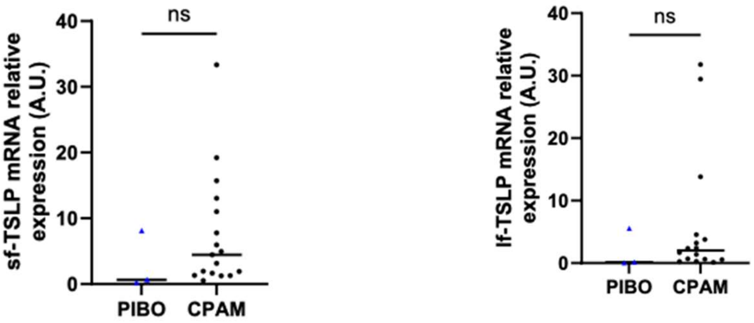

Figure S1. *Ex vivo* epithelium model functional characterization: analysis of alarmin isoforms in PIBO (blue dots) (n = 3) compared to controls (black dots) (n = 17).

Gene expression was measured in HBECs **A:** Short-form (sf) TSLP **B:** Long-form (lf) TSLP

Data are presented as median values in arbitrary units (A.U).

ns: not significant (Mann–Whitney U test)
